# Application of Seizon and Life Sciences to Global Public Health Challenges: An International Symposium

**DOI:** 10.31662/jmaj.2018-0020

**Published:** 2018-09-28

**Authors:** Hiroki Nakatani, Mari Michinaga, Hiroshi Kasanuki

**Affiliations:** 1 Keio-APRU Ageing Research Hub, Center for Integrated Medical Research, Keio University School of Medicine, Tokyo, Japan; 2 Japan Medical Association, Tokyo, Japan; 3 Waseda University, Tokyo, Japan

**Keywords:** Universal health coverage, Seizon Kagaku, Seizon and Life Sciences, Global Health, Takemi Program

## Abstract

The JMA HSPH Taro Takemi Memorial International Symposium “*Community Health Systems and Innovations: Building the Foundation for Universal Health Coverage*” was held on February 17, 2018. The symposium was co-organized by the Japan Medical Association, Harvard T.H. Chan School of Public Health, the Tokyo Medical Association, and Takemi Memorial Trust for Research of Seizon and Life Sciences. The aim of this symposium was to offer a forum for medical associations, academia, health policy makers, and other stakeholders to discuss the way forward to expand universal health coverage (UHC) in a rapidly changing environment surrounding health and human well-being, a concept that can be traced to the philosophy of late Dr. Taro Takemi. The three keynote lectures provided wide social and ethical as well as historic and global perspectives on health. They were followed by three sessions that each addressed one central theme: lessons learnt from the Japanese experience of responding to unprecedented demographic challenges (Session 1), how innovations can link national and global health policies with people’s well-being (Session 2), and how these efforts can be sustained (Session 3). Finally, a concluding lecture attempted to apply the philosophy of Dr. Taro Takemi, known as Seizon and Life Sciences, to UHC based on the discussions of the symposium. In our opinion, Dr. Taro Takemi’s foresight and philosophy should be revisited when we attempt to address present and future challenges; therefore, this symposium will be remembered for opening new ways of thinking.

The JMA HSPH Taro Takemi Memorial International Symposium “*Community Health Systems and Innovations: Building the Foundation for Universal Health Coverage*” was held on February 17, 2018 at the Japan Medical Association (JMA) Auditorium, Tokyo. The symposium was co-organized by the JMA, the Harvard T.H. Chan School of Public Health (HSPH), the Tokyo Medical Association, and the Takemi Memorial Trust for Research of Seizon and Life Sciences. The Executive Committee for the planning and execution of the symposium was chaired by Professor Hiroshi Kasanuki, and Dr. Mari Michinaga served as the Secretary General and Professor Hiroki Nakatani as the Deputy Chair. A summary of the report will appear on the website of the Takemi Memorial Trust for Research of Seizon and Life Sciences ^(1)^.

The concept of the meeting gradually emerged during the planning. The meeting was originally planned as an event commemorating the 35^th^ anniversary of the Takemi Program in International Health, which was founded at the Harvard School of Public Health in October 1983. Then, the theme of the symposium was expanded to include active follow-up of universal health coverage (UHC) included in Goal 3 of the United Nations Sustainable Development Goals, and contributions of JMA to UHC on the occasion of the election of Dr. Yoshitake Yokokura as the 68^th^ president of the World Medical Association in October 2017. The program of the symposium is depicted in Table 1.

**Table 1.** Program of the JMA HSPH Taro Takemi Memorial International Symposium.

**Opening** Mari Michinaga, Executive Board Member, JMA **Greeting** Yoshitake Yokokura, President of JMA, President of WMA Keizo Takemi, Member, Executive Committee; Member, House of Councillors **Congratulatory Remarks** Katsunobu Kato, Minister of Health, Labour and Welfare

**Keynote Lectures** Chaired by: Hiroki Nakatani, Executive Board Member, World Health Organization (WHO); Professor for Global Research Institute, Keio University **Lecture 1:** “Social Justice and Health Policy” Sir Michael Marmot, Past President of WMA, Professor in Epidemiology and Public Health, University College London (UCL) **Lecture 2:** “Moving Towards Universal Health Coverage, Step by Step” Michael Reich, Director, Taro Takemi Research Professor in International Health Policy, Takemi Program in International Health, Harvard T.H. Chan School of Public Health **Lecture 3:** “Contribution of the World Medical Association” Yoshitake Yokokura, President of JMA, President of WMA

**Session 1: Community Health and Healthy Ageing: Learning for Low Birthrate and Aging Society, Japan** Chaired by Keizo Takemi, Member, House of Councillors **Lecture 1**: “Promoting a Life Cycle Approach in Low Birthrate and Ageing Society” Aya Goto, Professor, Integrated Center for Science and Humanities, Fukushima Medical University **Lecture 2:** “Prevention of Lifestyle-Related Disease under the System of Community Medicine” Hiroyasu Iso, Professor, Public Health Graduate School of Medicine, Osaka University **Lecture 3:** “Contribution of Local Medical Association for Health Ageing and Transformation of Hospitals to Support Home Care and Community Care for Older People” Kunihiko Suzuki, Executive Board Member, JMA **Lecture 4:** “Primary Health Care for Achieving Universal Health Care for All and the Elderly in India” K Sujatha Rao, Former Secretary of the Ministry of Health and Family Welfare of India

**Special Lecture** Chaired by Haruo Ozaki, President of Tokyo Medical Association (TMA) “The Health Challenges of Preparing for the Olympic and Paralympic Games – Is it worth it? Lessons from London 2012” Brian McCloskey CBE, WHO Collaborating Centre for Mass Gatherings and Global Health Security, Public Health England, UK. “General Health Consequences of Olympic Games: Lessons from Rio Olympics and Paralympics” Marcia Castro, Associate Professor of Demography, Department of Global Health and Population, Harvard T.H. Chan School of Public Health

**Session 2: Linking Community and World through Innovation** Chaired by Hiroshi Kasanuki, University Professor, Waseda University **Lecture 1:** “IT Innovation / System / Social Innovation: PeOPLe” Hiroaki Miyata, Professor, Department of Health Policy and Management School of Medicine, Keio University **Lecture 2:** “Innovations for Comprehensive Prevention, Treatment, and Care for Cognitive Declined Citizens” Hidetaka Ota, Senior Specialist for Dementia, Office for Dementia Policy, Health and Welfare Bureau for the Elderly, Ministry of Health, Labour and Welfare **Lecture 3:** “Innovation in Long-Term Care Using ICT and Social Innovations” Tai Takahashi, Dean and Chairman, School of Health and Welfare, International University of Health and Welfare **Lecture 4:** “On the Frontier of Community Health: Advanced by Innovation and Constrained by Context” Jesse Bump, Executive Director, Takemi Program in International Health, Lecturer on Global Health Policy, Harvard T.H. Chan School of Public Health

**Session 3: Future Framework to support Community Health in Global Context** Chaired by Michael Reich, Director of Takemi Program, Taro Takemi Research Professor of International Health Policy, Harvard T.H. Chan School of Public Health **Lecture 1:** “Service Delivery for Universal Health Coverage” Mickey Chopra, Global Solutions Lead for Service Delivery, World Bank **Lecture 2:** “Paradigm Shift in Collaboration: Asia Human Wellbeing Initiative” Keizo Takemi, Member, House of Councillors **Lecture 3:** “Dynamism on International Coordination – To Improve Access to Pharmaceuticals and Medical Devices by Regulatory Authorities” Tatsuya Kondo, Chief Executive of the Pharmaceuticals and Medical Devices Agency (PMDA)

**Concluding Lecture and Reflections** Chaired by Mari Michinaga, Executive Board Member, JMA “Seizon Science as Foundation of UHC” Ryozo Nagai, President, Jichi Medical University

**Summing-up by Chairs of three Sessions:** Keizo Takemi, Hiroshi Kasanuki, and Michael Reich

## 

Dr. Yokokura’s opening speech set the tone for the symposium. As a general concept, UHC is important for the promotion of physical and mental health and well-being as well as to extend healthy life expectancy for all people. Practically, UHC is a vehicle to create innovative models, which guide countries for tackling unprecedented demographic changes with innovative and tailor-made solutions. Dr. Yokokura posed the question of what countries, medical associations, and physicians can do to achieve UHC individually and collectively. Indeed, the symposium was designed to provide a forum to discuss these issues based on Japanese experiences and from global perspectives.

The keynote lectures provided wide social and ethical as well as historic and global perspectives of health. They were followed by three sessions, each addressing one central theme: lessons learnt from the Japanese experience of responding to unprecedented demographic challenges (Session 1), how innovations can link national and global health policies with people’s well-being (Session 2), and how these efforts can be sustained (Session 3). Finally, a concluding lecture attempted to apply the philosophy of Dr. Taro Takemi, known as Seizon and Life Sciences, to UHC based on the discussions in the symposium. In addition, Tokyo will host the Olympic and Paralympic Games in 2020, and the countdown has already begun. Two speakers who served as the main advisors for health preparation in the London (2012) and Rio de Janeiro (2016) Games were invited to share their experiences.

Session 1, “*Community Health and Healthy Ageing: Learning for Low Birthrate and Ageing Society, Japan*” was chaired by Senator Keizo Takemi, Member of House of Councillors. After virtually realizing UHC in 1961, Japan has seen unprecedented demographic changes, with rapid population ageing and declining birthrates. In order to address the present and future challenges, the health systems that provide UHC have to be transformed. The four lectures investigated different aspects: (1) a life cycle approach for an ageing society with low birthrates, (2) the integration of public health and community health care for the prevention of lifestyle-related diseases, (3) the role of medical associations in transforming health system to support home and community care, and (4) reflection on emerging health care business in India. These presentations described how innovative attempts have been implemented in some districts in Japan with great success. They may serve as models for delivering UHC for countries that are undergoing the same demographic changes as Japan. The sessions also focused on efforts at the community level, which is a unique level where individual health care needs and national health policy framework meet. Senator Takemi concluded this session by inviting all participants to think about the future direction of UHC when we celebrate the 40^th^ anniversary of the Alma Ata Declaration of Health for All in October 2018. It is an appropriate time to reflect on the tremendous changes experienced during the past 40 years and on where UHC is heading to prepare us for the future.

Session 2, “*Linking Community and World through Innovation*,” was chaired by Professor Hiroshi Kasanuki, Waseda University. Four lectures highlighted how innovative technologies may revolutionize future health systems and services and how technology has changed communities. Three presentations focused on different domains wherein innovations are very active: (1) IT innovations that impact system and social innovations; (2) comprehensive prevention, treatment, and care for citizens experiencing cognitive decline; and (3) long-term care using ICT and social innovations. The fourth lecture looked at how technological innovations have completely changed the context of community health. IT can revolutionize the design, planning, and financing of health policy. Various innovations for people with dementia and seniors in need of long-term care have the potential to improve their QOL. However, such innovations need to be widely applied at a community level, but the synergy remains less than optimal. Therefore, it is necessary not only to apply further technological innovation to the lives of people in the community but also to create social innovations that optimize their applications.

Session 3, “*Future Framework to Support Community Health in Global Context*,” was chaired by Professor Michael Reich, Director of the Takemi Program at HSPH. This session aimed to link local experience and multisectoral innovations with their wider and systematic applications at regional, national, and global levels. The lectures covered (1) service delivery for UHC, (2) the Asia Health and Human Well-Being Initiative as an example of a paradigm shift in collaboration, and (3) improving access to pharmaceuticals and medical devices by regulatory authorities through international coordination. We learned that innovations have generally contributed to improve health; however, we should not be overly optimistic because, too often, one focuses on a relatively easy part of the system at the expense of building capability and governance of the whole system. One needs to pay attention to coherence across the system; otherwise, changing a system by innovation may cause more harm than good. Also, relationships between countries are shifting from vertical to horizontal collaboration. Horizontal collaboration is the spirit of the Asia Health and Human Well-Being Initiative, which invites Asian countries experiencing rapid population ageing with different timelines to mutually help each other. This mutuality represents a paradigm shift from the old donor–recipient relationship. This session concluded with the acknowledgement of a systematic approach to maximize the benefits of innovations for population health via improved UHC.

The concluding lecture and reflection session was chaired by Dr. Mari Michinaga, Executive Board Member of JMA. Professor Ryozo Nagai, the president of Jichi Medical University, discussed “*Seizon Science as the Foundation of UHC.*” Seizon and Life Sciences, or science and arts for human survival and well-being, is the philosophy advocated by Dr. Taro Takemi. It is an extremely interdisciplinary concept that integrates not only natural sciences but also social sciences and humanities, aiming for healthier and more humane survival for all people, individually and collectively. This concept stemmed from Dr. Takemi’s broad interest and understanding of medicine, biophysics, philosophy, ethics, economics, law, and politics.

In the form of summarizing the symposium, Prof. Nagai expressed his reflections on how UHC would relate to Dr. Taro Takemi’s ideas and philosophy. The life history of Dr. Takemi portrays an exceptional personality who was a medical practitioner, scientist, innovator, and policy maker. Dr. Takemi perceived that the fundamental weakness of medicine in Japan is its piecemeal approach, which looked only at the present illness, disassociating from the wider vision or philosophy of understanding human well-being in a holistic manner. This was the background of Seizon and Life Sciences, and Dr. Takemi posed many critical and practical questions relating to human well-being and survival. One of them was “development and redistribution of medical resources” in 1975. Another important philosophy of Dr. Takemi is “backcasting” as opposed to forecasting. He believed that if one looks at the present from the perspective of the future, one can separate the historical society from the biological society and can see the present more clearly while considering the social and natural environment of the future. When one studies his principle philosophy, one begins to see connections and integration with UHC and other aspects that were discussed in the symposium.

Finally, the three session chairs made a final statement of reflection and emphasized on the key points of the respective sessions. This unique one-day meeting started with discussions on past Japanese experiences with UHC and the challenges that we faced and overcame, followed by presentations on innovations, and finally the way forward to achieve UHC as a sustainable goal for Japan and the world.

Japan is facing an imminent population decline, with its growing super-ageing population and low birthrate. During this process, many issues will surely surface. One issue is financial constraints that will arise from trying to maintain the present level of generous health insurance coverage for a growing number of beneficiaries and to solve the health gaps within the nation. The sustainability of what we are proud of, the universal health insurance coverage that safeguards UHC, will be challenged. In the book *Collapse: How Societies Choose to Fail or Succeed*, Jared Diamond ^(2)^ raised two crucial choices that decide the failure or success of a society when it comes to survival: long-term planning and willingness to reconsider core values. The first choice refers to the courage to practice long-term thinking and to make bold, courageous, anticipatory decisions. The second choice is the courage to make painful decisions about values by asking which of the values that formerly served a society can be maintained under new, changed circumstances, and which of these treasured values must instead be jettisoned and replaced with different approaches. For Japan, the time for making these choices may come soon. Then, Dr. Taro Takemi’s foresight on the value of “backcasting” will once again be appreciated, and this symposium will be remembered for opening new ways of thinking.

## Article Information

### Conflicts of Interest

None

### Acknowledgement

The authors are thankful to the following executive committee members for their contributions in the whole process of the meeting; the names of members appear in alphabetical order.

Yoshiharu Aizawa, Masamine Jimba, Hiroyuki Kamano, Terukazu Kato, Katsuyuki Kinosita, Hideaki Koizumi, Tatsuya Kondo, Eiji Marui, Katsuhiko Mikoshiba, Ryozo Nagai, Haruo Naito, Haruo Ozaki, Michael Reich, Keizo Takemi, and Yoshitake Yokokura.

### Author Contributions

HN, MM, and HK organized the symposium. HN drafted the report. MM and HK revised the manuscript critically. HN, MM, and HK approved the submission of the manuscript.
